# Bleomycin Aggravates Atopic Dermatitis via Lung Inflammation in 2,4-Dinitrochlorobenzene-Induced NC/Nga Mice

**DOI:** 10.3389/fphar.2018.00578

**Published:** 2018-06-01

**Authors:** Yoon-Young Sung, Seung-Hyung Kim, Won-Kyung Yang, Yang-Chun Park, Ho Kyoung Kim

**Affiliations:** ^1^Herbal Medicine Research Division, Korea Institute of Oriental Medicine, Daejeon, South Korea; ^2^Institute of Traditional Medicine and Bioscience, Daejeon University, Daejeon, South Korea

**Keywords:** bleomycin, CD4+ cells, dinitrochlorobenzene, hyperresponsiveness, lung, pulmonary fibrosis

## Abstract

Atopic dermatitis (AD) is a chronic inflammatory skin disease. Bleomycin (BLM) contributes to the induction of pulmonary inflammation and fibrosis in animals. Although skin and lung tissue inflammation is closely related in the pathogenesis of allergic diseases, a proper animal model for investigating the relationship between skin and lung inflammation is lacking. Therefore, we developed a mouse model of AD with relapsing dermatitis and pulmonary fibrosis caused by the administration of allergen and BLM. The present study determined whether lung injury caused by the bronchial application of BLM would exacerbate AD-like allergic inflammation induced by 2, 4-dinitrochlorobenzene (DNCB) in NC/Nga mice. NC/Nga mice treated with BLM and DNCB had increased severity of clinical symptoms and airway hyperresponsiveness as well as increased inflammatory cell infiltration and collagen deposition in the dorsal skin and lung. Compared to normal mice, interleukin (IL)-6 and tumor necrosis factor (TNF)-α production in bronchoalveolar lavage fluid were increased in NC/Nga mice treated with both DNCB and BLM and in animals treated with DNCB alone. Administration of BLM and DNCB increased the levels of IL-4 and IL-13 production in spleen cells and eotaxin-2 mRNA expression in dorsal skin, compared to NC/Nga mice treated with DNCB alone. The total cell numbers in axillary lymph node, bronchoalveolar lavage, and thymus were increased in DNCB-BLM mice compared to those in mice treated with DNCB alone. Administration of BLM and DNCB increased the numbers of cluster of differentiation 4 (CD4)+ T cells and CD11b+granulocyte-differentiation antigen-1 (Gr-1)+ cells among peripheral blood mononuclear cells, CD4+ cells in bronchoalveolar lavage, CD4+ and B220+CD23+ B cells in the axillary lymph node, and CD4+ cells in thymus, compared to DNCB-treated mice. The number of total, CD4+, and CD11b+Gr-1+ cells in the lung were increased in both DNCB and DNCB-BLM mice. These results demonstrate that BLM aggravates allergic skin inflammation and promotes airway hyperreactivity and lung inflammation when combined with DNCB in NC/Nga mice.

## Introduction

Atopic dermatitis (AD) is a chronic inflammatory skin disease that has a high prevalence among infants and children (Han et al., 2011). The pathogenesis of AD is strongly influenced by both genetic and environmental factors (Bantz et al., 2014). The skin, located on the outermost body surface, is an important interface between the host and its environment, and the skin is believed, in Oriental medicine, to be influenced by the physiological function of the lung on the body (Im et al., 2002; Glick, 2005). In Asia, AD is called ‘skin asthma,’ because so many infants and children with AD already have, or will develop, asthma (Glick, 2005). Development of AD in infancy, and the subsequent development of other allergic respiratory diseases such as asthma in later childhood is referred to as the atopic march (Spergel and Paller, 2003; Zheng et al., 2011). Some studies have developed animal models with localized AD induced by repeated epicutaneous sensitization with ovalbumin, and airway hyperresponsiveness to methacholine after challenge with aerosolized ovalbumin, to study the progression from AD to asthma (Spergel et al., 1998). Recently, Han et al. (2017) reported that neonatal capsaicin treatment induced chronically relapsing pruritic dermatitis and asthmatic airway changes in rats (Han et al., 2017). However, a proper animal model for investigating the relationship among skin and lung inflammation in AD is still lacking.

Bleomycin (BLM) administration is a widely used method for inducing local skin and pulmonary inflammation and fibrosis in animals (Liang et al., 2015). Intratracheal instillation of BLM in mice causes bronchial fibrotic changes, acute interstitial and intra-alveolar inflammation, and upregulation of alveolar inflammatory cells (Della Latta et al., 2015). C57BL/6 mice, which are more susceptible than Balb/c mice to BLM-induced fibrosis, are used in most studies, and only few researchers employ other strains such as 129, CBA, Balb/c, and ICR (Della Latta et al., 2015). We developed a mouse model of AD with relapsing dermatitis and pulmonary fibrosis by the administration of BLM and allergen. The present study determined whether lung injury induced by the bronchial application of BLM would exacerbate the AD-like allergic inflammation induced by 2, 4-dinitrochlorobenzene (DNCB) in NC/Nga mice, an inbred animal model for human AD (Choi et al., 2012; Kim et al., 2014). It has been reported that repeated applications of DNCB to the skin of NC/Nga mice induced AD-like skin lesions, which is associated with an increase in serum immunoglobulin (Ig) E and T-helper (Th) 2 cytokines such as interleukin (IL)-4, IL-5, and IL-13 at the chronic dorsal skin lesions (Choi et al., 2012). In addition, NC/Nga mice showed high airway hyperresponsiveness to acetylcholine and marked enhancement of airway resistance after a single intranasal challenge with ovalbumin or mite allergen *Dermatophagoides farinae*, thus suggesting that NC/Nga mice are a useful animal model to investigate the pathogenesis of allergic asthma and AD (Iwasaki et al., 2001; Shibamori et al., 2006).

Therefore, the purpose of this study was to develop an animal model for studying AD in mice using timely administration of BLM and DNCB. We induced AD by repeated topical application of DNCB to the skin after induction of lung fibrosis by bronchial injection of BLM in NC/Nga mice. In the present study, we have demonstrated, using a murine model of AD, that bleomycin, when administered in combination with allergens, has an additive and synergistic effect in driving cutaneous eczematoide skin changes, and promotes airway hyperreactivity and lung inflammation upon allergen challenge.

## Materials and Methods

### Animals

Five-week-old male NC/Nga and C57BL/6 mice were obtained from Central Lab Animal Inc. (Seoul, South Korea). All animal experiments and procedures were approved by the Committee for Animal Welfare at the Daejeon University (DJUARB2016-036). All animal procedures were conducted in accordance with the guidelines of the Animal Care and Use Committee of the South Korea Research Institute of Bioscience and Biotechnology (Daejeon, South Korea) and the US guidelines (NIH publication number 85-23. Revised 1996). All animals were housed in air-conditioned animal room at a temperature of 21 ± 2°C and humidity of 50 ± 5% under a 12:12-h light/dark cycle and had *ad libitum* access to food and water for 1 week.

### Induction of Lung Fibrosis

The mice were divided into four groups (*n* = 4): group 1, C57BL/6 (vehicle)-Normal; group 2, NC/Nga (vehicle)-Normal; group 3, NC/Nga (DNCB)-Control; group 4, NC/Nga (DNCB-BLM). As previously described (Della Latta et al., 2015), the NC/Nga mice were administered a single-dose intratracheal instillation of bleomycin (BLM-treated group, 0.125 U/100 g; Sigma) or vehicle (normal and control group, saline) using bronchial tubes. Normal C57BL/6 mice received vehicle only.

### Induction of AD

**FIGURE 1:**
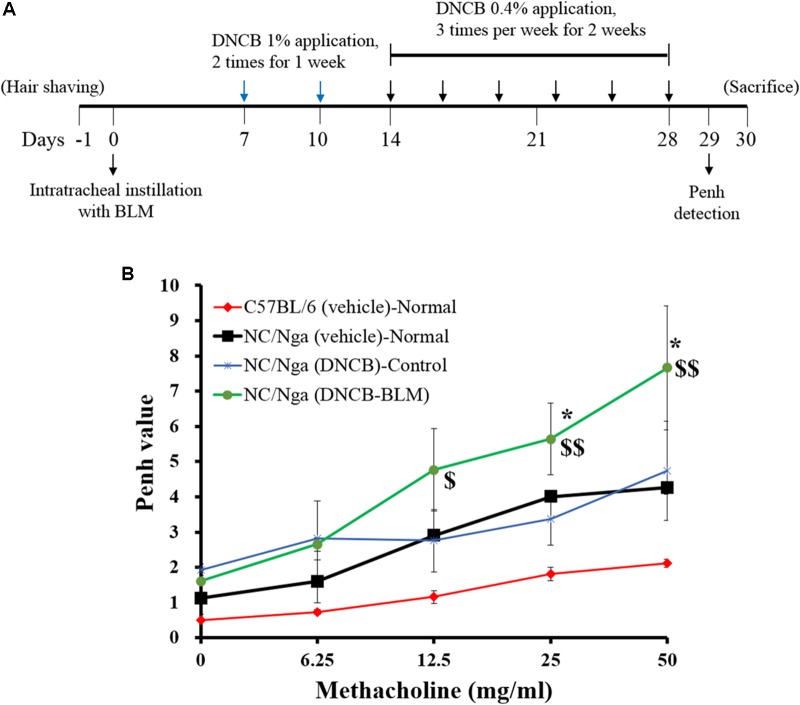
Effect of BLM on airway hyperresponsiveness. **(A)** Schematic diagram of the treatment protocol. **(B)** Airway resistance was measured after exposure to increasing doses of aerosolized methacholine (6.25, 12.5, 25, and 50 mg/ml). The mice were divided into four groups (*n* = 4); C57BL/6 (vehicle)-Normal, NC/Nga (vehicle)-Normal, NC/Nga (DNCB)-Control, and NC/Nga (DNCB-BLM). Values are expressed as the mean ± standard error of mean (SEM) (*n* = 4). ^$^*p* < 0.05, ^$$^*p* < 0.01 [compared with C57BL/6 (vehicle)-Normal], and ^∗^*p* < 0.05 [compared with NC/Nga (DNCB)-Control] as determined by one-way ANOVA followed by multiple comparison tests.

The back hair of mice was shaved with electric clippers a day before BLM administration. AD was induced in C57BL/6 and NC/Nga mice 7 days after BLM or vehicle administration (Park et al., 2014; Saba et al., 2016). For the sensitization process, 200 μl of a 1% DNCB solution (acetone: olive oil = 3:1) was applied dermally to the shaved back area twice per week for 1 week using 1 × 1 cm patches. At 1 week after the first DNCB sensitization, the back skin was challenged with 150 μl of a 0.4% DNCB solution three times per week for 3 weeks. At the end of the experiment, mice were sacrificed by intraperitoneal (i.p.) injection of urethane (2.5 mg/kg), and samples were collected. The schematic diagram of the experimental protocol is shown in **Figure 1A**.

### Collection of Bronchoalveolar Lavage Fluid (BALF)

After the mice were sacrificed by i.p. injection of urethane (2.5 mg/kg), BALF was collected by washing the airway lamina by tracheal cannulation. The fluid collected from airway lavage was defined as BALF, centrifuged, and the supernatant collected and stored at -25°C for determination of cytokine levels. The cell pellets were suspended in 1 ml PBS and the total cell number was counted using a hemocytometer.

### Determination of Airway Hyperresponsiveness (AHR)

Airway hyperresponsiveness in mice was measured using non-invasive whole-body plethysmography Buxco system (Biosystems XA; DSI Inc., MN, United States) as previously described (Kim et al., 2011). Each chamber was equipped with a pneumotachograph (Halcyon, Buxco) to measure airflow, and this information was transmitted to analysis software (Biosystem XA for windows, Buxco). The software uses various algorithms to calculate several flow-derived parameters, including respiratory rate, lung volume, peak flow, and time intervals. The software reported data as ‘enhanced pause’ (Penh), an index of airway hyperreactivity derived from the equation: Penh is equal to Pause × PEF/PIF, where Pause = (Te-Tr)/Tr (PIF, peak inspiratory flow; PEF, peak expiratory flow; Te, expiratory time; Tr, relaxation time). One day after the final DNCB treatment, the mice were placed in Buxco chamber and were given aerosolized normal saline, followed by increasing doses (6.25, 12.5, 25, and 50 mg/ml) of aerosolized methacholine (Sigma-Aldrich Korea, Seoul, South Korea). Airway reactivity was then monitored for 30 min and respiratory curves were converted into enhanced pause (Penh) values.

### Measurement of Clinical Skin Severity Score

Dermatitis skin scores of AD-like skin lesions were evaluated as described previously (Suto et al., 1999). The severity of dermatitis was assessed according to four symptoms: (1) erythema/hemorrhage, (2) scarring/dryness, (3) edema, and (4) excoriation/erosion. Each symptom was scored from 0 to 3 (none, 0; mild, 1; moderate, 2; severe, 3). Clinical skin severity score was defined as the sum of the individual scores, ranging from 0 to 12.

### Enzyme-Linked Immunosorbent Assay (ELISA)

The levels of IgE in the serum were determined using a mouse ELISA kit (R&D Systems, Minneapolis, MN, United States). Interleukin (IL)-6 and TNF-α secretion in BALF were determined by ELISA (R&D systems) according to the manufacturer’s instructions.

IL-4, IL-5, IL-13, and IFN-γ production was detected in spleen cells suspended in RPMI 1640 medium supplemented with 2 mM L-glutamine and 5% FBS. Spleen cells were cultured in 96-well plates for 48 h at a concentration of 1 × 10^5^ cells/well with plate-bound anti-CD3 antibody (0.5 μg/ml) in a humidified atmosphere of 5% CO_2_ at 37°C. The culture supernatants were then collected and IL-4, IL-5, IL-13, and IFN-γ levels in the supernatants were measured using an ELISA kit (R&D systems).

The limits of detection (sensitivity) were 1.8 pg/ml of IL-6, 7.21 pg/ml of TNF-α, 2 pg/ml of IL-4, 7.0 pg/ml of IL-5, 1.5 pg/ml of IL-13, and 2 pg/ml of IFN-γ. According to the certificate of analysis supplied by the manufacturer of the commercial assay, the intra-assay coefficient of variation (CV) of the IL-4 ELISA system was 4.7% and the inter-assay CV was 5.2%. The intra- and inter-assay CV of IL-5 was 5.2 and 4.4%, respectively. The intra- and inter-assay CV of IL-13 was 2.8 and 6.7%, respectively. The intra-and inter-assay CV of IFN-γ was 3.6 and 9.3%, respectively. The intra- and inter-assay CV of IL-6 was 4.7 and 7.5%, respectively. The intra- and inter-assay CV of TNF-α was 3.2 and 7.7%, respectively.

### Sample Collection and Cell Preparations

Peripheral blood mononuclear cells (PBMCs) were isolated from the heparinized blood of mice by Percoll density-gradient centrifugation. Cell suspensions were prepared from fresh tissues processed in RPMI 1640 medium. Single cell suspensions from tissues and BALF were isolated by mechanical disruption in RPMI 1640 medium supplemented with 2 mM L-glutamine, 100 U/mL penicillin, 100 μg/mL streptomycin, 50 μM 2-mercaptoethanol, 20 mM HEPES, and 2% heat-inactivated fetal bovine serum (FBS, GIBCO, Grand Island, NY, United States). Briefly, the tissues (axillary lymph node, thymus, dorsal skin, and lung) were removed from the mice, minced using scalpels, and then incubated in PBS containing 1 mg/mL collagenase IV (Sigma-C5138, Sigma, St. Louis, MO, United States) at 37°C. After incubation for 40 min, each tissue was vigorously pipetted up and down to further dissociate the remaining tissue clumps. The cell suspension was filtered using a 70-μm pore size nylon cell strainer (BD Falcon, Bedford, MA, United States) and then centrifuged for 20 min at 450 g. The cell pellet was collected, and the cells were washed twice. Total cell number was determined using a hemocytometer chamber (Thermo Fisher Scientific, Grand Island, NY, United States). The cells obtained were stained immediately with various antibodies for flow cytometry analysis.

### Flow Cytometry Analysis

Prior to flow cytometry analysis, samples abundant with RBCs were treated with ammonium-chloride-potassium (ACK) lysis buffer for 2 min at room temperature. PBMCs, as well as cells from the axillary lymph node (ALN), BAL, thymus, dorsal skin, and lung tissue were stained with the indicated antibodies in staining solution (PBS containing 1% FBS and 0.01% NaN3) for 10 min on ice. After the samples were stained, the stained cells were assessed on a fluorescence-activated cell sorter flow cytometry system (FACS Calibur; BD Biosciences, Mountain View, CA, United States) and the data were analyzed with CellQuest software (BD Biosciences). All antibodies, including anti-CD4, anti-B220, anti-CD11b, anti-Gr-1, anti-CD25, and anti-CD23 were purchased from BD PharMingen (San Diego, CA, United States). The absolute number of each cell subtype was calculated from the subtype percent and the total number of cells in the tissues of each individual mouse.

### Hematoxylin-Eosin (H&E), Masson’s Trichrome (MT), and Toluidine Blue (TB) Staining

Dorsal skin and lung tissue were removed and subjected to histological analysis using a previously published protocol (Kim et al., 2011; Sung et al., 2014). Tissues was fixed in 10% (v/v) neutral-buffered formalin, embedded in paraffin, and then cut into 3-μm thick sections. Dorsal skin and lung sections were stained with H&E to assess the infiltration of inflammatory cells and epidermal hyperplasia. To determine the degree of pathologic changes (epidermal and dermal hypertrophy and inflammation), index counts extent of epidermal/dermal thickness and inflammatory cells in the dorsal skin lesions were blindly quantified with subjective scores of 0 (none), 1 (mild), 2 (moderate), and 3 (severe) for each of the four symptoms using a previously published protocol with modification (Kang et al., 2015). Other sections were stained with TB to evaluate the infiltration of mast cells in the dorsal skin, and with MT solution (Sigma-Aldrich Korea) to determine collagen deposition in the lung. At least three different sections of each tissue were evaluated under light microscopy.

### Quantitative Real-Time Polymerase Chain Reaction (RT-qPCR)

Dorsal skin tissues from the mice were removed and immediately frozen by immersion in liquid nitrogen for subsequent RNA isolation. Total RNA from the dorsal skin was extracted using RNAzol B (Tel-Test, Austin, TX, United States) according to the manufacturer’s instructions. The cDNA was synthesized from 3 μg of total RNA using a ReverTra Ace-a-cDNA Synthesis kit (Toyobo, Osaka, Japan). The cDNA template was mixed with SYBR Green PCR Master Mix (Applied Biosystems, Grand Island, NY, United States) and 200 nM primers. Glyceraldehyde-3-phosphate dehydrogenase (GAPDH) was used as an internal control. Quantitative real-time RT-PCR was performed in triplicate using the Applied Biosystems 7500 Fast Real-Time PCR system (Applied Biosystems) and analyzed according to the manual (threshold: 0.05, baseline: 6–15 cycles). PCR was performed under the following set of conditions: 2 min at 50°C, 10 min at 94°C, 40 cycles of 1 min at 94°C, and 1 min at 60°C. The cycle number at which the emission intensity of the sample rose above baseline was defined as the relative quantity (RQ) and was proportional to the target concentration. The RQ in target gene expression relative to control mice was normalized to the internal control, glyceraldehyde 3-phosphate dehydrogenase (GAPDH), using the 2^-ΔΔ^*^C^*^t^ method. The primer sequences were as follows: GAPDH, sense 5′-TGAAGCAGGCATCTGAGGG-3′, antisense 5′-CGAAGGTGGAAGAGTGGGAG-3′; IL-31R, sense 5′-ATGCCCAACAAAGCAGAGAC-3′, antisense 5′-TGAGAGAACCAGGGAGCTGT-3′; and eotaxin-2, sense 5′- CTGTGACCATCCCCTCATCT-3′, antisense 5′-CTTATGGCCCTTCTTGGTGA-3′.

### Statistical Analysis

Results were expressed as mean ± standard error of the mean (SEM). Statistical analyses of the results were performed by one-way ANOVA followed by Tukey’s or Duncan’s multiple comparison tests using the SigmaPlot 13.0 software. Significance level was set at *p* < 0.05.

## Results

### Induction of Airway Hyperresponsiveness by Bleomycin

To determine the effects of BLM on lung fibrosis and function, we analyzed airway resistance in response to methacholine (doses of 6.25, 12.5, 25, and 50 mg/kg) by non-invasive WBP (**Figure 1B**). Pehn values for NC/Nga (vehicle) and NC/Nga (DNCB) mice were similar. However, Pehn values showed a greater increase in NC/Nga mice treated with both DNCB and BLM (DNCB-BLM) than in NC/Nga mice treated with DNCB alone (DNCB-Control) at doses of 25 (*F*-value = 5.470 and *p* = 0.017) and 50 mg/kg methacholine (*F*-value = 4.012 and *p* = 0.041).

### DNCB-Induced AD-Like Symptoms

**FIGURE 2:**
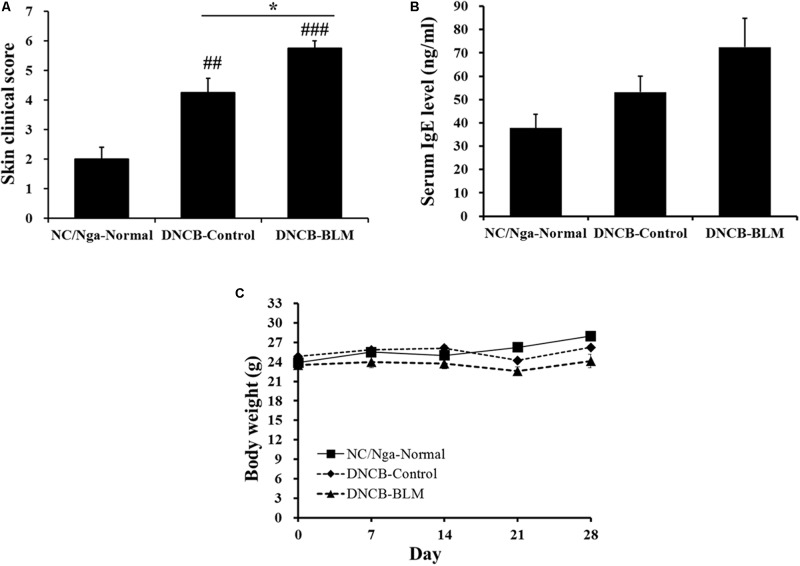
Effect of BLM on AD-like symptoms, serum IgE level, and body weight. **(A)** The severity of clinical symptoms of the skin lesions was evaluated macroscopically and calculated as the sum of the individual scores for the following four AD signs and symptoms: erythema/hemorrhage, edema, excoriation/erosion, and scaling/dryness. **(B)** Total IgE levels in serum were determined by ELISA. **(C)** Body weight were measured once a week. Values are expressed as the mean ± standard error of mean (SEM) (*n* = 4). ^##^*p* < 0.01, ^###^*p* < 0.001 (compared with NC/Nga-Normal), and ^∗^*p* < 0.05 (compared with DNCB-Control) as determined by one-way analysis of variance (ANOVA) followed by multiple comparison tests.

To determine whether BLM treatment in the lung exacerbates AD-like symptoms in DNCB-induced NC/Nga mice, we treated mice with DNCB and BLM. AD-like symptoms were evaluated by dermatitis skin score (Method 2.6) and the skin clinical score significantly increased in both DNCB-Control and DNCB-BLM mice when compared to NC/Nga-Normal mice (*F*-value = 19.696 and *p* ≤ 0.01) (**Figure 2A**). Consistent with previous reports (Choi et al., 2017), the overall clinical symptoms of AD, including erythema, hemorrhage, edema, scarring, excoriation, and lichenification, were induced by the repeated application of DNCB to the back skin of the NC/Nga mice for 2 weeks (*p* = 0.003). More importantly, BLM administered to the lung dramatically increased the skin clinical severity score of AD in DNCB-treated mice (*p* ≤ 0.001, compared to NC/Nga-Normal mice; *p* = 0.049, compared to DNCB-Control mice) (**Figure 2A**). In addition, although it did not achieve significance, the serum IgE concentration showed a greater increase in mice treated with DNCB and BLM than in mice treated with DNCB alone (**Figure 2B**). The body weight, in accordance with the worsening of AD symptoms, showed a tendency to decline in DNCB-treated or DNCB-BLM-treated mice; however, this reduction was not statistically significant (**Figure 2C**).

### Total Leukocyte Cell Numbers From the ALN, Dorsal Skin, BALF, Thymus, and Lung

**FIGURE 3:**
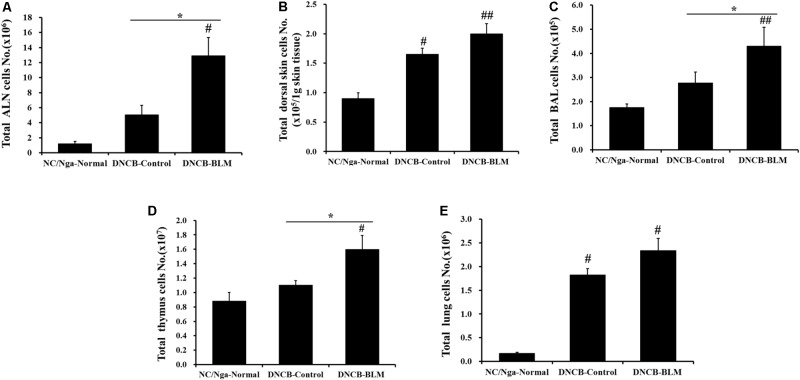
Effect of BLM on total cell numbers in the **(A)** ALN, **(B)** dorsal skin, **(C)** BAL, **(D)** thymus, and **(E)** lung. Values are expressed as the mean ± standard error of mean (SEM) (*n* = 4). ^#^*p* < 0.05, ^##^*p* < 0.01 (compared with NC/Nga-Normal), and ^∗^*p* < 0.05 (compared with DNCB-Control) as determined by one-way analysis of variance (ANOVA) followed by multiple comparison tests.

Next, we investigated whether BLM treatment in the lung in AD mice altered the total number of leukocytes in ALN, dorsal skin, BAL, thymus, and lung. Total ALN cells were significantly higher in DNCB-BLM-treated mice than in the NC/Nga-Normal (*F* = 8.842 and *p* = 0.015) and DNCB-control groups (*p* = 0.040) (**Figure 3A**). Total BAL cells were significantly higher in DNCB-BLM-treated mice than in the NC/Nga-Normal (*F* = 3.54 and *p* = 0.005) and DNCB-control groups (*p* = 0.011) (**Figure 3C**). Total thymus cells were significantly higher in DNCB-BLM-treated mice than in the NC/Nga-Normal (*F*-value = 5.521 and *p* = 0.036) and DNCB-control groups (*p* < 0.05) (**Figure 3D**). Our analysis demonstrated a statistically significant increase in total cell numbers of dorsal skin in both DNCB-Control (*F*-value = 9.6 and *p* ≤ 0.05) and DNCB-BLM mice (*p* < 0.01) when compared to NC/Ng-Normal mice (**Figure 3B**). Total lung cells were significantly higher in DNCB-Control and DNCB-BLM mice compared to NC/Nga mice (*F*-value = 22.403 and *p* ≤ 0.05, **Figure 3E**); the values in dorsal skin and lung cells were significantly higher in the DNCB-BLM-treated mice than in NC/Nga-Normal mice (*p* < 0.05), but there was no significant difference between DNCB-Control and DNCB-BLM-treated mice (**Figures 3B,E**).

### Absolute Number of Immune Cell Subtypes

**FIGURE 4:**
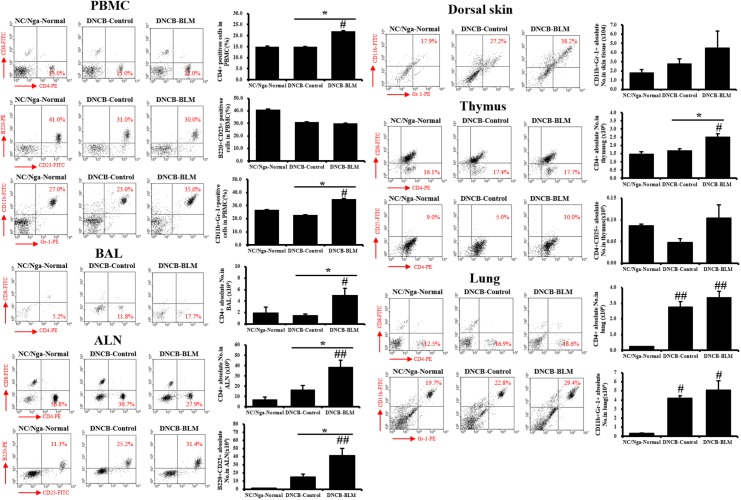
Immune cell subtypes in PBMCs, BAL, ALN, dorsal skin, thymus, and lung. The cell subtypes were analyzed by fluorescence-activated cell sorting analysis (FACS). Red numbers on the scatter plots indicate the percentage of each cell type in the total immune cell population. The absolute number of each cell type was calculated from the specific cell type percentage and total cell number counted in the tissues of each individual mouse. Values are expressed as the mean ± standard error of mean (SEM) (*n* = 4). ^#^*p* < 0.05, ^##^*p* < 0.01 (compared with NC/Nga-Normal), and ^∗^*p* < 0.05 (compared with DNCB-Control) as determined by one-way analysis of variance (ANOVA) followed by multiple comparison tests.

To confirm whether the AHR and inflammatory changes observed in mice subjected to various treatments were reflected in altered T cell subtypes and B cell distributions in PBMCs, BAL, ALN, dorsal skin, thymus, and lung, flow cytometry analysis was performed (**Figure 4**).

The DNCB-BLM-treated mice displayed a statistically significant higher number of CD4+ helper T cells in PBMCs (*F*-value = 4.872 and *p* = 0.047), BAL (*F*-value = 4.823 and *p* = 0.048), ALN (*F*-value = 7.286 and *p* = 0.002), thymus (*F*-value = 10.93 and *p* = 0.044), and lung (*F*-value = 15.61 and *p* = 0.003) compared to NC/Nga-Normal mice. In addition, CD4+ helper T cells in the PBMCs, BALF, ALN, and thymus were significantly increased in the DNCB-BLM-treated mice when compared to DNCB-Control mice (*p* < 0.05). IgE-producing B220+CD23+ B cells in the ALN were higher in DNCB-BLM-treated mice than in NC/Nga-Normal (*F*-value = 8.000 and *p* = 0.002) and DNCB-Control (*p* = 0.022) mice, but were unchanged in PBMCs. In PBMC, CD11b+Gr-1+ granulocytes showed a statistically significant increase (*F*-value = 4.120 and *p* ≤ 0.05) in the DNCB-BLM-treated mice when compared to NC/Nga-Normal mice. Further, the number of CD11b+Gr-1+ granulocytes in the PBMC were significantly elevated in the DNCB-BLM-treated mice when compared to DNCB-Control (*p* = 0.04). In lung, CD11b+Gr-1+ granulocytes showed a statistically significant increase (*F*-value = 8.364 and *p* < 0.05) in the DNCB-Control and DNCB-BLM-treated mice when compared to NC/Nga-Normal mice. In the dorsal skin, CD11b+Gr-1+ cells were increased in the DNCB-BLM mice, but the differences were not significant among the groups. CD4+CD25+ regulatory T cells in the thymus also showed no significant differences among the three groups.

### Increase in Cytokine Production in BALF and Splenocyte Culture Supernatant

**FIGURE 5:**
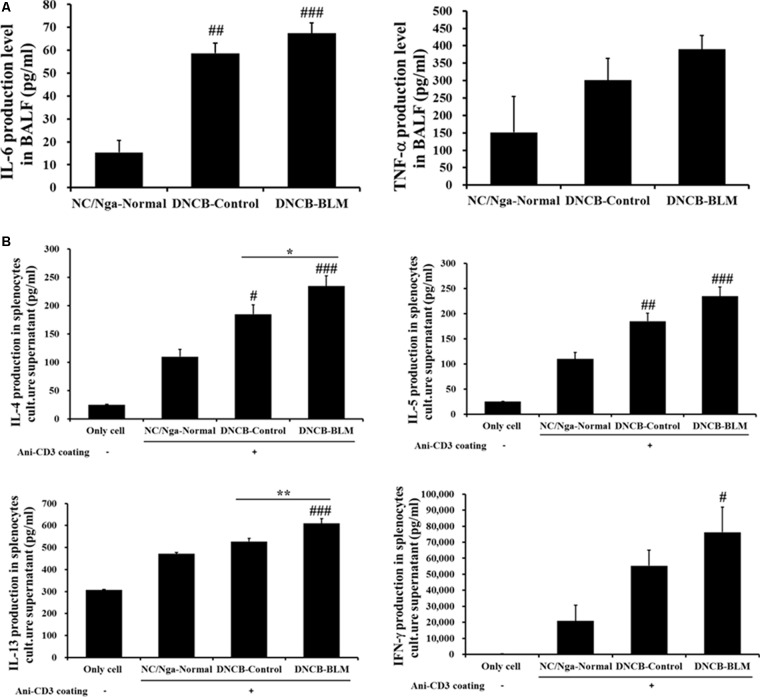
Effect of BLM on the production of pro-inflammatory cytokines (IL-6 and TNF-α) in BALF **(A)**, and Th2 cytokines (IL-4, IL-5, and IL-13) and Th1 cytokines (IFN-γ) production in spleen cells **(B)**. Spleen cells were cultured for 48 h at a concentration of 1 × 10^5^ cells/well using anti-CD3 coated 96-well plates. The ‘Only cell’ group represents the negative control (i.e., no exposure to anti-CD3 antibody). Cytokine production was measured by ELISA. Values are expressed as the mean ± standard error of mean (SEM) (*n* = 4). ^#^*p* < 0.05, ^##^*p* < 0.01, ^###^*p* < 0.001 (compared with NC/Nga-Normal), ^∗^*p* < 0.05, ^∗∗^*p* < 0.01 (compared with DNCB-Control) as determined by one-way analysis of variance (ANOVA) followed by multiple comparison tests.

To investigate whether BLM influenced pro-inflammatory cytokine secretion in BALF, the levels of IL-6 and TNF-α were measured by ELISA. The differences in IL-6 levels in BALF between the groups were statistically significant (*F*-value = 24.351 and *p* ≤ 0.001). As shown in **Figure 5A**, IL-6 levels were significantly increased in the DNCB-Control (*p* = 0.002) and DNCB-BLM-treated mice (*p* < 0.001) when compared to the NC/Nga-Normal mice. TNF-α levels in BALF were increased in the DNCB-Control and DNCB-BLM-treated mice, but this difference was not statistically significant across the groups. The differences in IL-4 (*F*-value = 10.113 and *p* ≤ 0.001), IL-5 (*p* ≤ 0.001), IL-13 (*F* = 11.731 and *p* =≤ 0.001), and IFN-γ (*p* = 0.033) levels in spleen cell culture supernatants between the groups were statistically significant. Our data indicate that DNCB-BLM treatment resulted in a dramatic increase in the Th1 cytokine IFN-γ (*p* < 0.05), as well as in the Th2 cytokines IL-4 (*p* < 0.001), IL-5 (*p* < 0.001), and IL-13 (*p* < 0.001) compared to NC/Nga-Normal mice. These levels in DNCB-BLM mice were higher than those observed in DNCB-Control mice (*p* < 0.05 for IL-4 and *p* < 0.01 for IL-13) (**Figure 5B**).

### Histopathological Analysis of Dorsal Skin and Lung Tissues

**FIGURE 6:**
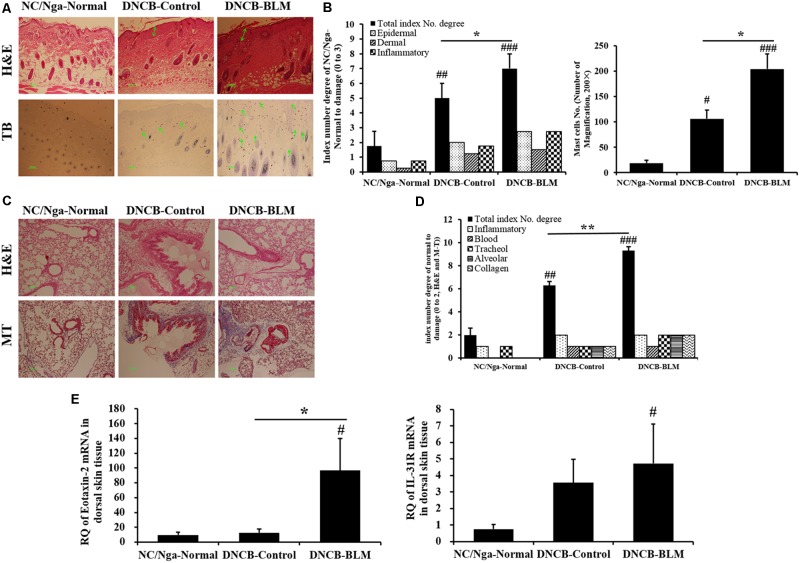
Histopathological changes in dorsal skin and lung tissues, and expression of eotaxin-2 and IL-31R mRNA in dorsal skin. **(A)** Skin sections were stained with H&E and TB. **(B)** Total index number and mast cell number in the dorsal skin were quantitated. **(C)** Lung sections were stained with H&E and MT. **(D)** Total index number of degree in the lung was quantified. **(E)** Expression levels of eotaxin2 and IL-31R mRNA in dorsal skin tissue were analyzed by real-time RT-PCR. The results are expressed as RQ to control. H&E, hematoxylin-eosin stain; TB, toluidine blue stain; MT, Masson trichrome stain; RQ, relative quantity. Values are expressed as the mean ± standard error of mean (SEM) (*n* = 4). ^#^*p* < 0.05, ^##^*p* < 0.01, ^###^*p* < 0.001 (compared with NC/Nga-Normal), ^∗^*p* < 0.05, ^∗∗^*p* < 0.01 (compared with DNCB-Control) as determined by one-way analysis of variance (ANOVA) followed by multiple comparison tests.

To investigate the effects of BLM on histopathological changes in the DNCB-induced AD model, dorsal skin tissues were stained with H&E and TB staining solution. **Figures 6A,B** demonstrate the differences among the groups, which were also found to be statistically significant (*F*-value = 28.886 and *p* =≤ 0.001). The staining results showed that the dorsal skin of DNCB-Control mice exhibited epidermal hypertrophy, inflammatory cell infiltration (*p* = 0.003), and increased mast cell numbers (*p* < 0.05). These inflammatory and mast cell number changes were worse in the DNCB-BLM mice (*p* < 0.05).

In addition, we performed H&E and MT staining of lung tissue sections to evaluate lung inflammation and collagen identification. Collagen content has been used as a measure of the degree of fibrosis in the lung (McAnulty and Laurent, 1995); these measurements were made in the lungs of DNCB- or DNCB-BLM-treated mice. BLM increased inflammatory infiltrate and collagen deposition in the peribronchial and perivascular areas of DNCB-BLM mice (*F* = 73.4, *p* < 0.001 compared with NC/Nga-Normal mice, *p* = 0.006 compared with DNCB-Control mice; **Figures 6C,D**).

### Increase of Eotaxin-2 and IL-31R mRNA in Dorsal Skin Tissues

To determine the effects of BLM on the gene expression levels of allergy-related biomarkers in dorsal skin tissue, we measured the expression of eotaxin-2 and IL-31R mRNA by real-time RT-PCR. The results showed that mRNA expression of eotaxin-2 was dramatically increased in the DNCB-BLM mice compared to that in NC/Nga-Normal and DNCB-Control mice (*p* < 0.05, **Figure 6E**). Expression of IL-31R mRNA was significantly higher in DNCB-BLM mice compared to that in NC/Nga mice (*p* < 0.05), and the levels were not significantly different between DNCB-Control and DNCB-BLM mice (**Figure 6E**).

## Discussion

We developed a mouse model of AD induced by DNCB-BLM treatment. In the present study, we showed in NC/Nga mice that, when combined with allergens DNCB, BLM has an additive effect on driving AD-like symptoms and promotes asthmatic changes such as airway hyperreactivity and allergic lung inflammation. Taken together, this model could be useful for studying the pathogenesis and progression of allergic atopic diseases. Mice that received both DNCB and BLM showed AD-like symptoms including erythema, edema, hemorrhage, scarring, and excoriation. DNCB and BLM treatment increased the protein production of Th2 cytokines (such as IL-4, IL-5, and IL-13) and IFN-γ in spleen cells, and of IL-6 and TNF-α in BALF. Th2 lymphocytes play a prominent role in the initiation and progression of allergic disease, including asthma and AD, by releasing IL-4, IL-5, and IL-13, which promote IgE-mediated allergic inflammation, and IFN-γ, a Th1 cytokine secreted by Th1 cells, acts in conjunction with Th2 cytokines in maintaining the chronic inflammatory response in allergic disease, particularly in asthmatic airways (Ngoc et al., 2005). IL-4 causes a switch to IgE production by differentiating B cells, and elevated eosinophilic inflammation is under the control of IL-5 and TNF-α (Deo et al., 2010). IL-13 directly induces mucus hypersecretion and airway hyperresponsiveness (Kim et al., 2011). Furthermore, changes of cytokines in BALF reflect the immunological reaction of the lung in asthma and other inflammatory pulmonary diseases. Together with TNF-α, IL-6 as a pro-inflammatory cytokine plays a major role in dictating the immune response against allergens, as well as in specific pathological features of allergic airway inflammation such as mucus production (Neveu et al., 2009).

Eotaxin-1, -2, and -3 are potent and specific eosinophil chemoattractants involved in the activation and recruitment of eosinophils through binding to their CC chemokine receptor 3. Thus, eotaxins might contribute to increasing blood and tissue eosinophils in allergic inflammation responses (Kaplan, 2001). Eotaxin-1 is an important mediator involved in the early phase of allergic inflammation via allergen-induced recruitment of eosinophils to sites of allergic inflammation, and eotaxin-2 and eotaxin-3 play a prominent role in the subsequent persistence of allergen-induced skin or bronchial eosinophilia (Ravensberg et al., 2005). Eotaxin-2 and eotaxin-3 are produced by bronchial epithelial cells in response to Th2 cytokines such as IL-4 and IL-13 (Komiya et al., 2003), which might suggest that these chemokines could be secreted at sites of Th2-dominant allergic inflammation. Studies demonstrate that the levels of eotaxin and IL-5 in skin of patients with AD were higher than those of healthy controls, and that these molecules are involved in the proliferation, recruitment, activation, and survival of eosinophils in AD (Park et al., 2005). The present study demonstrated that mRNA expression levels of eotaxin-2 are increased in dorsal skin lesions of mice that received both DNCB and BLM. The results indicate that increased eotaxin-2 and IL-5 expression in mice that received both DNCB and BLM contribute to eosinophil-mediated inflammation in AD.

IL-31 is a cytokine produced mainly by activated CD4+ Th2 cells or by mast cells, and is involved in the development of AD-induced skin inflammation and pruritus (Saito et al., 2017). IL-31, a pruritogenic cytokine, signals via a heterodimeric receptor composed of IL-31 receptor A and oncostatin M receptor β, which is expressed by a number of cell types, including epidermal keratinocytes, epithelial cells, mast cell, eosinophils, and activated monocytes and macrophages (Cheung et al., 2010). The IL-31 receptor (IL-31R) was expressed at higher levels on epidermal keratinocytes in skin biopsy specimens from AD patients, compared to those from healthy individuals (Bilsborough et al., 2006). The anti-IL-31 receptor α subunit neutralizing antibody reduced ear swelling and dermatitis score in a chronic AD model in BALB/c mice (Kasutani et al., 2014). The present study showed that expression of IL-31R mRNA was increased in dorsal skin lesions of mice that received both DNCB and BLM compared to those of normal mice. But, IL-31R mRNA levels were not significantly different between DNCB-Control and DNCB-BLM mice. These results indicate that increased IL-31R expression by DNCB-BLM treatment might contribute to the development of AD-induced skin inflammation and pruritus.

The present study showed that DNCB-BLM treatment induced an increase in total leukocytes and specific CD4+ helper T cells in the ALN, BALF, and thymus of the mice. The CD4+ T cell is a dominant activated T cell subtype in clinical allergic asthma and in animal models of asthma, an mainly contributes to AHR development and allergic inflammation by secreting Th2 cytokines such as IL-4, IL-5, and IL-13 (Serre et al., 2010; Raemdonck et al., 2016). Moreover, B220+CD23+ B cells were increased in the ALN of DNCB-BLM-treated mice. CD23 is a low-affinity receptor for IgE (Fc𝜀RII) which is expressed on B cells, monocytes, T cells, dendritic cells, and neutrophils, and the CD23 surface density on antigen-presenting B cells is associated with IgE levels as well as the activation of allergen-specific T cells (Selb et al., 2017). DNCB-BLM treatment increased B cell-dependent production of total IgE in serum by approximately twofold compared to that in normal mice, albeit without statistical significance; this finding correlates with the observation of increased B220+CD23+ B cells in the ALN of mice that received both DNCB and BLM. CD11b is a cell surface molecule that is highly expressed in eosinophils, and its expression on the eosinophil surface is increased in allergic diseases including bronchial asthma and AD (Yachie et al., 1993). Gr-1 is a granulocytic marker that is related to the differentiation and maturation of granulocytes including eosinophils, basophils, and neutrophils (Fleming et al., 1993). Eosinophils are one of the cell types known to express Gr-1; therefore, eosinophils may constitute a substantial portion of the CD11b+Gr-1+ population (Kim et al., 2011). Our results showed that CD11b+Gr-1+ cells were increased in PBMCs and in the lungs of DNCB-BLM-treated mice; this increase in CD11b+Gr-1+ cells may contribute to development of allergic inflammation.

## Conclusion

The present study demonstrated the development of an animal model for studying AD in mice using timely administration of bleomycin and DNCB. This study demonstrates that bleomycin, when administrated in combination with DNCB, aggravates allergic skin inflammation and promotes airway hyperreactivity and lung inflammation, in NC/Nga mice. These findings indicate that this bioassay has potential as a tool to use in furthering our understanding of AD, as well as a screen for irritants and treatments in future studies.

## Author Contributions

Y-CP and HK contributed to the conceptualization and design of the work. S-HK performed the animal experiment. W-KY carried out the analysis of animal samples. Y-YS contributed the analysis and interpretation of data. Y-YS wrote the manuscript. All authors approved the final version to be published.

## Conflict of Interest Statement

The authors declare that the research was conducted in the absence of any commercial or financial relationships that could be construed as a potential conflict of interest.

## References

[B1] Bantz S. K. Zhu Z. Zheng T. (2014). The atopic march: progression from atopic dermatitis to allergic rhinitis and asthma. *J. Clin. Cell. Immunol.* 5:202. 10.4172/2155-9899.1000202 25419479 PMC4240310

[B2] Bilsborough J. Leung D. Y. Maurer M. Howell M. Boguniewicz M. Yao L. (2006). IL-31 is associated with cutaneous lymphocyte antigen-positive skin homing T cells in patients with atopic dermatitis. *J. Allergy Clin. Immunol.* 117 418–425. 16461143 10.1016/j.jaci.2005.10.046

[B3] Cheung P. F. Wong C. K. Ho A. W. Hu S. Chen D. P. Lam C. W. (2010). Activation of human eosinophils and epidermal keratinocytes by Th2 cytokine IL-31: implication for the immunopathogenesis of atopic dermatitis. *Int. Immunol.* 22 453–467. 10.1093/intimm/dxq027 20410259

[B4] Choi J. H. Park B. H. Kim H. G. Hwang Y. P. Han E. H. Jin S. W. (2012). Inhibitory effect of *Psidium guajava* water extract in the development of 2,4-dinitrochlorobenzene-induced atopic dermatitis in NC/Nga mice. *Food Chem. Toxicol.* 50 2923–2929. 10.1016/j.fct.2012.04.044 22609491

[B5] Choi J. H. Song Y. S. Lee H. J. Kim G. C. Hong J. W. (2017). The topical application of low-temperature argon plasma enhances the anti-inflammatory effect of Jaun-ointment on DNCB-induced NC/Nga mice. *BMC Complement. Altern. Med.* 17:340. 10.1186/s12906-017-1850-9 28655324 PMC5488426

[B6] Della Latta V. Cecchettini A. Del Ry S. Morales M. A. (2015). Bleomycin in the setting of lung fibrosis induction: from biological mechanisms to counteractions. *Pharmacol. Res.* 97 122–130. 10.1016/j.phrs.2015.04.012 25959210

[B7] Deo S. S. Mistry K. J. Kakade A. M. Niphadkar P. V. (2010). Role played by Th2 type cytokines in IgE mediated allergy and asthma. *Lung India* 27 66–71. 10.4103/0970-2113.63609 20616938 PMC2893428

[B8] Fleming T. J. Fleming M. L. Malek T. R. (1993). Selective expression of Ly-6G on myeloid lineage cells in mouse bone marrow. RB6-8C5 mAb to granulocyte-differentiation antigen (Gr-1) detects members of the Ly-6 family. *J. Immunol.* 151 2399–2408. 8360469

[B9] Glick S. (2005). Treating childhood eczema with acupuncture and Chinese herbs. *Acupunct. Today* 6 1–8.

[B10] Han N. R. Kim H. M. Jeong H. J. (2011). Kanamycin activates caspase-1 in NC/Nga mice. *Exp. Dermatol.* 20 659–663. 10.1111/j.1600-0625.2011.01291.x 21569102

[B11] Han R. T. Kim S. Choi K. Jwa H. Lee J. Kim H. Y. (2017). Asthma-like airway inflammation and responses in a rat model of atopic dermatitis induced by neonatal capsaicin treatment. *J. Asthma Allergy* 10 181–189. 10.2147/JAA.S124902 28572736 PMC5441677

[B12] Im G. M. Jeong H. W. Kim H. S. Heong W. Y. (2002). Oriental medical approach on the allergic disease. *Korean J. Orient. Physiol. Pathol.* 16 831–839.

[B13] Iwasaki T. Tanaka A. Itakura A. Yamashita N. Ohta K. Matsuda H. (2001). Atopic NC/Nga mice as a model for allergic asthma: severe allergic responses by single intranasal challenge with protein antigen. *J. Vet. Med. Sci.* 63 413–419. 11346176 10.1292/jvms.63.413

[B14] Kang H. Lee C. H. Kim J. R. Kwon J. Y. Seo S. G. Han J. G. (2015). Chlorella vulgaris attenuates dermatophagoides farinae-induced atopic dermatitis-like symptoms in NC/Nga mice. *Int. J. Mol. Sci.* 16 21021–21034. 10.3390/ijms160921021 26404252 PMC4613239

[B15] Kaplan A. P. (2001). Chemokines, chemokine receptors and allergy. *Int. Arch. Allergy Immunol.* 124 423–431. 11340325 10.1159/000053777

[B16] Kasutani K. Fujii E. Ohyama S. Adachi H. Hasegawa M. Kitamura H. (2014). Anti-IL-31 receptor antibody is shown to be a potential therapeutic option for treating itch and dermatitis in mice. *Br. J. Pharmacol.* 171 5049–5058. 10.1111/bph.12823 24946165 PMC4253455

[B17] Kim H. Kim J. R. Kang H. Choi J. Yang H. Lee P. (2014). 7,8,4′-Trihydroxyisoflavone attenuates DNCB-induced atopic dermatitis-like symptoms in NC/Nga mice. *PLoS One* 9:e104938. 10.1371/journal.pone.0104938 25170825 PMC4149428

[B18] Kim S. H. Kim B. K. Lee Y. C. (2011). Antiasthmatic effects of hesperidin, a potential Th2 cytokine antagonist, in a mouse model of allergic asthma. *Mediators Inflamm.* 2011:485402. 10.1155/2011/485402 21772663 PMC3136080

[B19] Komiya A. Nagase H. Yamada H. Sekiya T. Yamaguchi M. Sano Y. (2003). Concerted expression of eotaxin-1, eotaxin-2, and eotaxin-3 in human bronchial epithelial cells. *Cell. Immunol.* 225 91–100. 14698143 10.1016/j.cellimm.2003.10.001

[B20] Liang M. Lv J. Zou L. Yang W. Xiong Y. Chen X. (2015). A modified murine model of systemic sclerosis: bleomycin given by pump infusion induced skin and pulmonary inflammation and fibrosis. *Lab. Invest.* 95 342–350. 10.1038/labinvest.2014.145 25502178

[B21] McAnulty R. J. Laurent G. J. (1995). “Collagen and its regulation in pulmonary fibrosis,” in *Pulmonary Fibrosis*, eds Phan S. H. Thrall R. S. (New York, NY: Marcel Dekker Inc), 135–171.

[B22] Neveu W. A. Allard J. B. Dienz O. Wargo M. J. Ciliberto G. Whittaker L. A. (2009). IL-6 is required for airway mucus production induced by inhaled fungal allergens. *J. Immunol.* 183 1732–1738. 10.4049/jimmunol.0802923 19592651 PMC2929571

[B23] Ngoc P. L. Gold D. R. Tzianabos A. O. Weiss S. T. Celedón J. C. (2005). Cytokines, allergy, and asthma. *Curr. Opin. Allergy Clin. Immunol.* 5 161–166. 15764907 10.1097/01.all.0000162309.97480.45

[B24] Park B. K. Park Y. C. Jung I. C. Kim S. H. Choi J. E. Park S. (2014). Oral administration of SSC201, a medicinal herbal formula, suppresses atopic dermatitis-like skin lesions. *J. Med. Food* 17 496–504. 10.1089/jmf.2013.2941 24476223 PMC3993068

[B25] Park C. W. Lee B. H. Han H. J. Lee C. H. Ahn H. K. (2005). Tacrolimus decreases the expression of eotaxin, CCR3, RANTES and interleukin-5 in atopic dermatitis. *Br. J. Dermatol.* 152 1173–1181 10.1111/j.1365-2133.2005.06474.x 15948978

[B26] Raemdonck K. Baker K. Dale N. Dubuis E. Shala F. Belvisi M. G. (2016). CD4+ and CD8+ T cells play a central role in a HDM driven model of allergic asthma. *Respir. Res.* 17:45. 10.1186/s12931-016-0359-y 27112462 PMC4845490

[B27] Ravensberg A. J. Ricciardolo F. L. van Schadewijk A. Rabe K. F. Sterk P. J. Hiemstra P. S. (2005). Eotaxin-2 and eotaxin-3 expression is associated with persistent eosinophilic bronchial inflammation in patients with asthma after allergen challenge. *J. Allergy Clin. Immunol.* 115 779–785. 15805998 10.1016/j.jaci.2004.11.045

[B28] Saba E. Lee C. H. Jeong da H. Lee K. Kim T. H. Roh S. S. (2016). Fermented rice bran prevents atopic dermatitis in DNCB-treated NC/Nga mice. *J. Biomed. Res.* 30 334–343. 10.7555/JBR.30.2016K0001 27323667 PMC4946324

[B29] Saito S. Aoki A. Arai I. Takaishi S. Ito H. Akiyama N. (2017). Regulation of Th2 responses by different cell types expressing the interleukin-31 receptor. *Allergy Asthma Clin. Immunol.* 13:23. 10.1186/s13223-017-0194-9 28428802 PMC5392993

[B30] Selb R. Eckl-Dorna J. Neunkirchner A. Schmetterer K. Marth K. Gamper J. (2017). CD23 surface density on B cells is associated with IgE levels and determines IgE-facilitated allergen uptake, as well as activation of allergen-specific T cells. *J. Allergy Clin. Immunol.* 139 290.e–299.e. 10.1016/j.jaci.2016.03.042 27372566 PMC5321593

[B31] Serre K. Mohr E. Gaspal F. Lane P. J. Bird R. Cunningham A. F. (2010). IL-4 directs both CD4 and CD8 T cells to produce Th2 cytokines *in vitro*, but only CD4 T cells produce these cytokines in response to alum-precipitated protein *in vivo*. *Mol. Immunol.* 47 1914–1922. 10.1016/j.molimm.2010.03.010 20392496 PMC3826121

[B32] Shibamori M. Ogino K. Kambayashi Y. Ishiyama H. (2006). Intranasal mite allergen induces allergic asthma-like responses in NC/Nga mice. *Life Sci.* 78 987–994. 10.1016/j.lfs.2005.06.020 16229861

[B33] Spergel J. M. Mizoguchi E. Brewer J. P. Martin T. R. Bhan A. K. Geha R. S. (1998). Epicutaneous sensitization with protein antigen induces localized allergic dermatitis and hyperresponsiveness to methacholine after single exposure to aerosolized antigen in mice. *J. Clin. Invest.* 101 1614–1622. 9541491 10.1172/JCI1647 PMC508742

[B34] Spergel J. M. Paller A. S. (2003). Atopic dermatitis and the atopic march. *J. Allergy Clin. Immunol.* 112 S118–S127. 14657842 10.1016/j.jaci.2003.09.033

[B35] Sung Y. Y. Lee A. Y. Kim H. K. (2014). The *Gardenia jasminoides* extract and its constituent, geniposide, elicit anti-allergic effects on atopic dermatitis by inhibiting histamine *in vitro* and *in vivo*. *J. Ethnopharmacol.* 156 33–40. 10.1016/j.jep.2014.07.060 25153023

[B36] Suto H. Matsuda H. Mitsuishi K. Hira K. Uchida T. Unno T. (1999). NC/Nga mice: a mouse model for atopic dermatitis. *Int. Arch. Allergy Immunol.* 129 70–75. 10.1159/000053599 10529609

[B37] Yachie A. Toma T. Miyawaki T. Taniguchi N. (1993). Expression of surface CD11b antigen and eosinophil activation. *Nippon Rinsho* 51 593–597. 8098378

[B38] Zheng T. Yu J. Oh M.H. Zhu Z. (2011). The atopic march: progression from atopic dermatitis to allergic rhinitis and asthma. *Allergy Asthma Immunol. Res.* 3 67–73. 10.4168/aair.2011.3.2.67 21461244 PMC3062798

